# Retraction Note: HMGB1: a novel protein that induced platelets active and aggregation via Toll-like receptor-4, NF-κB and cGMP dependent mechanisms

**DOI:** 10.1186/s13000-018-0747-3

**Published:** 2018-09-12

**Authors:** Xinyu Yang, Haichao Wang, Menmen Zhang, Jin Liu, Ben Lv, Fangping Chen

**Affiliations:** 1 Depatment of Haematology, The Third Xiangya Hospital, Central South University, Changsha, Hunan People’s Republic of China; 2 Laboratory of Emergency Medicine, The Feinstein Institute for Medical Research, North Shore-LIJ Health System, Manhasset, NY 11030 USA; 3 Department of Hemotology Xiangya Hospital, Central South University, Changsha, Hunan 410078 People’s Republic of China

## Retraction Note: Yang et al. BMC Diagnostic Pathology (2015) 10:134 DOI 10.1186/s13000-015-0348-3

This article [1] is retracted at request of the Editor. After publication of this article [1] concerns were raised regarding the methodology, in particular the controls being insufficient to provide data to fully support the stated conclusions. In addition, there was inadequate documentation of the statistical methods used. Furthermore, errors in the language throughout the text and poor labeling of figures were potentially misleading to the reader. The authors have been given an opportunity to submit a corrected version. All authors agree to this retraction. Author Haichao Wang has stated that he was not involved in the preparation and submission of the above manuscript.
